# Development of Ciprofloxacin-Loaded Electrospun Yarns of Application Interest as Antimicrobial Surgical Suture Materials

**DOI:** 10.3390/pharmaceutics16020220

**Published:** 2024-02-03

**Authors:** Jorge Teno, Maria Pardo-Figuerez, Zoran Evtoski, Cristina Prieto, Luis Cabedo, Jose M. Lagaron

**Affiliations:** 1 R&D Department, Bioinicia S.L., 46980 Paterna, Spain; 2 Novel Materials and Nanotechnology Group, Institute of Agrochemistry and Food Technology (IATA), Spanish Council for Scientific Research (CSIC), 46980 Paterna, Spain; mpardo@iata.csic.es (M.P.-F.); zoran.evtoski@iata.csic.es (Z.E.); cprieto@iata.csic.es (C.P.); 3 Polymers and Advanced Materials Group (PIMA), School of Technology and Experimental Sciences, Universitat Jaume I (UJI), 12006 Castellón, Spain; lcabedo@uji.es

**Keywords:** PHBVs, electrospinning, suture yarn, antimicrobial activity

## Abstract

Surgical site infections (SSI) occur very frequently during post-operative procedures and are often treated with oral antibiotics, which may cause some side effects. This type of infection could be avoided by encapsulating antimicrobial/anti-inflammatory drugs within the surgical suture materials so that they can more efficiently act on the site of action during wound closure, avoiding post-operative bacterial infection and spreading. This work was aimed at developing novel electrospun bio-based anti-infective fibre-based yarns as novel suture materials for preventing surgical site infections. For this, yarns based on flying intertwined microfibres (1.95 ± 0.22 µm) were fabricated in situ during the electrospinning process using a specially designed yarn collector. The electrospun yarn sutures (diameter 300–500 µm) were made of poly(3-hydroxybutyrate-co-3-hydroxyvalerate) with different contents of 3HV units and contained ciprofloxacin hydrochloride (CPX) as the antimicrobial active pharmaceutical ingredient (API). The yarns were then analysed by scanning electron microscopy, Fourier transform infrared spectroscopy, wide-angle X-ray scattering, differential scanning calorimetry, and in vitro drug release. The yarns were also analysed in terms of antimicrobial and mechanical properties. The material characterization indicated that the varying polymer molecular architecture affected the attained polymer crystallinity, which was correlated with the different drug-eluting profiles. Moreover, the materials exhibited the inherent stiff behaviour of PHBV, which was further enhanced by the API. Lastly, all the yarn sutures presented antimicrobial properties for a time release of 5 days against both Gram-positive and Gram-negative pathogenic bacteria. The results highlight the potential of the developed antimicrobial electrospun yarns in this study as potential innovative suture materials to prevent surgical infections.

## 1. Introduction

Sutures are a key component in every surgical procedure that requires bringing together damaged tissues, thereby promoting healing after an injury, ligation of blood vessels, and hemostasis, among others [1,2,3]. The materials used in sutures and their manufacturing processes have improved over the years, generating multiple suture options depending on the site of action, depth, tension, type of material, and mechanical strength [3]. However, little has been pursued to enhance their therapeutic effect in treating wounds locally to avoid surgical site infections (SSI) [4]. SSI occurs quite often post-operatively and is normally treated with oral antibiotics before and after surgery, which may lead to unnecessary side effects. Additionally, if oral therapy fails, the infection can spread, and microorganisms can grow on the sutures, hindering the wound healing process whilst migrating and infecting adjacent tissues [5,6]. Thus, it is necessary to incorporate antimicrobial/anti-inflammatory drugs into the sutures to create high local drug concentrations without excessive systemic levels to efficiently act on the site of action [7].

The incorporation of antimicrobial drugs into the sutures has been frequently achieved using melt spinning [8], dip coating [9], layer by layer [6], or soaking procedures, among others [9,10]. A study reported by Wang et al. [11] used a commercial silk fibre previously coated with sequential layers of poly(allylamine hydrochloride), dextran, and hyaloplasm acid. Thereafter, the samples were soaked in an aqueous solution of ibuprofen to obtain the final drug-eluting material. The release studies showed that up to 76% of the loaded ibuprofen was released within 24 h [11]. Although effective, these types of techniques do not appropriately encapsulate the component of interest and consequently control their release kinetics, and their stability over time can become a challenge.

Electrospinning has been used to produce nano- and microfibrous structures that can efficiently incorporate drugs within a polymeric matrix in a single step [1,12,13]. This technique is based on the use of voltage in a polymeric solution that enables the formation of electrospun fibres, which are deposited into a collector [14]. The versatility of this technique allows for the deposition of the materials in a wide range of collector geometries, leading to the generation of planar materials such as mats or 3D-like structures such as conduits [15] or yarns [16]. Regarding the latter, a modification of the collector setup can generate a continuous thread-like structure made of nano- and microfibres that may mimic the structure of a yarn [16]. This type of material presents many advantages compared to other yarn manufacturing processes [1] and may be key to replacing the traditional threads in terms of properties and functional performance. For instance, electrospun fibres can encapsulate active components within their structure, providing a localized, controlled, and reliable drug delivery compared to other drug-eluting manufacturing processes, such as soaking or dip coating procedures. Additionally, electrospinning does not require high processing temperatures, so the technique could generate sutures that encapsulate thermolabile APIs such as proteins, peptide growth factors, DNA, or other sensitive pharmaceutical compounds that would not be feasible when using melt spinning. The use of electrospinning has been recently used to generate sutures, proving its potential for this application. As an example, Kashiwabuchi et al. [17] developed and manufactured electrospun sutures composed of poly(L-lactide), polyethylene glycol (PEG), and levofloxacin for ophthalmic surgery. The authors achieved a sustained release of the antibiotic for months as well as a strong bacterial zone inhibition of S. epidermidis after 7 days in release media. PLLA is one of the gold standard biopolymers used for biomedical applications; however, other biopolymers such as polylactide glycolide (PLGA) [18], polydioxanone (PDS) [19] polylactide (PLA), [6,20] and polyhydroxyalkanoates (PHAs) [20,21] can also be used as innovative materials to generate sutures.

PHAs are a family of biodegradable and highly biocompatible materials that have already proven successful in biomedical applications [22]. Although plenty of PHAs have been discovered, the most commonly investigated are poly(3-hydroxybutyrate) (PHB) and its copolymer poly(3-hydroxybutyrate-co-3-hydroxyvalerate) (PHBV). PHB has high crystallinity and macromolecular organization, resulting in a stiff and brittle material that lacks mechanical strength [23]. On the contrary, the co-polyester PHBV shows improved thermal and mechanical properties, which vary with the content of 3HV units present in the polyester. In fact, a higher 3HV content implies lower crystallinity and broader thermal processing, resulting in a more flexible, ductile, and tough material to work with [23]. Hence, PHBV has become an attractive candidate for biomedical applications [23,24]. Currently, commercial PHBV is limited to 3HV contents of 2 mol%, which present properties similar to those of commercial PHB grades. For this reason, new materials with different 3HV units are being synthesized and investigated to achieve a better balance in properties for numerous applications [23].

The present study was aimed at the development of antibiotic drug-eluting PHBV yarns prepared by electrospinning using a custom-designed funnel collector to prevent SSI. For this, PHBVs with different 3HV contents (2, 10, and 20 mol%) and ciprofloxacin hydrochloride (CPX) were selected as the material and drug antimicrobial models for yarn development, respectively. CPX is an antibiotic belonging to the family of fluoroquinolones, with a broad antibacterial activity against Gram-negative and Gram-positive bacteria [25]. The studies performed on electrospun yarns included microscopic, thermal, and mechanical characterization to unveil the surface morphology and physical and mechanical features of the materials. To determine the suitability of the material as a drug-eluting material, in vitro release studies of CPX and antimicrobial properties were carried out for the different CPX-PHBVs manufactured.

## 2. Materials and Methods

### 2.1. Materials

Ciprofloxacin hydrochloride (CPX) was obtained from Uquifa (Castellbisbal, Spain). The PHBV2 (ENMAT Y1000P) was purchased from Tianan Biologic Materials (Ningbo, China) and delivered in the form of pellets. According to the manufacturer, the 3HV fraction of the commercial copolyester is 2 mol%. The polyester PHBV10 with a percentage of 10 mol% of 3HV content was kindly provided by Venvirotech SL (Santa Perpètua de Mogoda, Spain), and the PHBV20 with 20 mol% of 3HV was produced at Universidade NOVA de Lisboa (Lisbon, Portugal) using mixed microbial cultures (MMCs) as detailed in previous works [26]. The solvent 2,2,2-trifluoroethanol (TFE, ≥99%) was purchased from Alfa Aesar^TM^ (Karlsruhe, Germany). All the polymers and reagents were used as received without further purification.

### 2.2. Electrospun Fibre Yarn

For reader clarification throughout the text, we will use the term “electrospun fibre” when we refer to the individual ultrathin fibres that the yarns are fully made of. Similarly, we will refer to “electrospun yarn” when we describe such an entire bundle of electrospun fibres.

**Figure 1:**
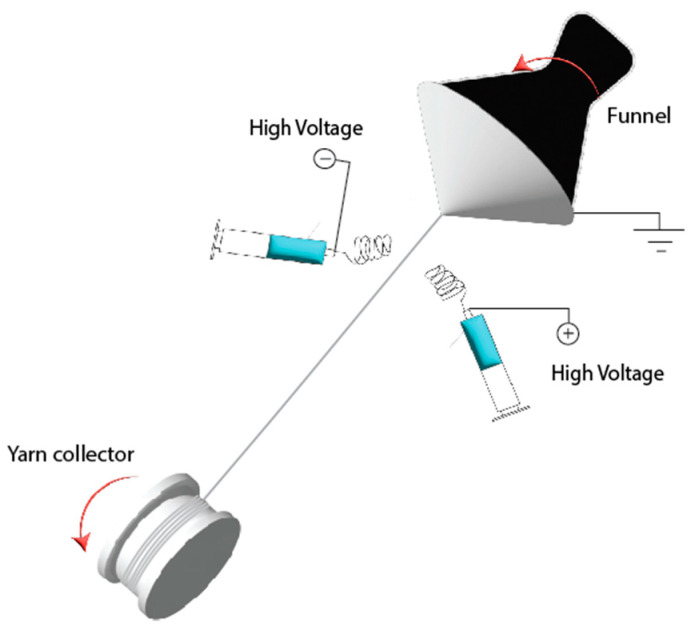
Schematics of the electrospun yarn-producing setup.

Prior to yarn production, different PHBVs solutions were prepared by dissolving each biopolymer in 8 wt.% in TFE. The CPX was added at 20 wt.% in relation to the polymer. This relationship was optimal for electrospinning, ensuring the complete dissolution of CPX. Solutions were prepared by adding the corresponding amounts of polymer and CPX to the solvent under stirring overnight at 37 °C. All polymer solutions were processed in a Fluidnatek^®^ LE-500 pilot-plant tool coupled with an air-conditioned unit system manufactured by Bioinicia S.L. (Valencia, Spain) adapted with a specifically engineered electrospun yarn-producing setup (See Figure 1 for the schematics of the equipment and Supplementary Material for a video of the setup running). Two similar spinning solutions (blue syringes in the schematic) were passed through two oppositely charged needle injector systems using an injection pump. The polymer was exerted on both sides of the setup and formed fibres under the action of a high-voltage electric field. The fibres with opposite charges coming from the two sides neutralized their charges and bundled together in the air by the force of the rotating funnel (black funnel) into yarns. The first thread of the fibre-based yarn was directed by tweezers to the motorized yarn collector reel placed on the other side of the equipment. The conditions for yarn production were set as shown in Table 1. Each solution was electrospun in a controlled environment at 30 °C and 30% RH. The collected sutures were maintained at room temperature in a desiccator at 0% RH until further analysis.

### 2.3. Yarn Characterization

#### 2.3.1. Morphology

The PHBV electrospun fibre yarns with and without CPX were examined by scanning electron microscopy (SEM) using a Phenom XL G2 Desktop microscope (Thermo Fisher Scientific, Waltham, MA, USA) with an electron beam acceleration of 5 kV. Samples were previously sputtered with a gold-palladium mixture for 3 min under vacuum. The average fibre diameter based on at least 100 fibres was determined using the Phenom ProSuite Software (https://www.phenom-world.com (accessed on 4 January 2024)) on SEM images.

#### 2.3.2. Wide-Angle X-ray Scattering (WAXS)

Wide-angle X-ray scattering measurements were performed using a Bruker AXS D4 Endeavor diffractometer. The samples were scanned at room temperature in reflection mode using incident Cu K-alpha radiation (Cu Kα = 1.54 Å), while the generator was set up at 40 kV and 40 mA. The data were collected over a range of scattering angles (2Θ) in the 5–40° range.

#### 2.3.3. Attenuated Total Reflection—Fourier Transform Infrared Spectroscopy (ATR-FTIR)

The presence of the CPX in the polymer matrix was evaluated by Fourier-transformed infrared spectroscopy and measured using a Bruker Tensor 37 FT-IR Spectrometer (Bruker, Ettlingen, Germany) coupled with the ATR sampling accessory Golden Gate (Specac Ltd., Orpington, UK). Spectra were collected from an average of 64 scans in the range of 4000–600 cm^−1^, with a resolution of 4 cm^−1^.

#### 2.3.4. Thermal Analysis

The electrospun fibre yarns were studied by differential scanning calorimetry (DSC) using a DSC-8000 analyser from PerkinElmer, Inc. (Waltham, MA, USA), equipped with a cooling accessory Intracooler 2 also from PerkinElmer, Inc. Approximately 3 mg of each sample were placed in standard aluminum pans and heated from 25 to 250 °C at a rate of 10 °C/min using a nitrogen flow of 20 mL/min as the sweeping gas.

#### 2.3.5. Mechanical Tests

The mechanical properties were determined using a universal testing machine (Shimadzu AGS-X 500 N) at room temperature with a cross-head speed of 10 mm/min and an initial distance between clamps (L0) of 25 mm. This method was based on the ASTM D638 standard. Tensile modulus (E), tensile strength (σ_b_), and elongation at break (ε_b_) were calculated from the stress–strain curves. Yarns specimens (n = 5) of 40 mm in length were tested.

#### 2.3.6. In Vitro Drug Release: Kinetics Study

The drug release was evaluated using a UV-spectrophotometer DINKO UV4000 (Barcelona, Spain). The release of CPX-loaded yarns (0.5 mg approx.) into an aqueous phosphate-buffered solution (pH 7.4) medium (150 mL) was monitored by measuring the absorbance at 270 nm at predetermined times whilst stirring at 100 rpm at 37 °C. At each time point, a 1 mL sample of the solution was extracted for UV–vis analysis for up to 96 h (4 days), and an equivalent volume of fresh buffer solution was introduced to uphold sink conditions. All the experiments were carried out in triplicate, and the cumulative release rate was reported as the mean ± S.D.

To determine the experimental loading of CPX within the fibre yarns, ca. 3 mg of CPX-loaded yarns were dissolved in 60 mL of TFE to fully dissolve both CPX and polymers. The resulting solution was stirred at 100 rpm for an hour at 37 °C and then analysed using a UV-spectrophotometer DINKO UV4000 (Barcelona, Spain). The reading was compared against a calibration curve produced using standard samples of 1–8 µg/mL CPX in TFE.

The absorbances at 270 nm were recorded, and the experimental CPX loading (%) in the fibres was calculated using the following equation:

(1) $$\mathrm{CPX}\;\mathrm{Loading}\left(\%\right)=\frac{m_{d}}{m_{p}}\times 100$$

where m_d_ is the mass of the drug obtained experimentally as described above, and m_p_ is the total mass of the sample analysed.

Additionally, the semi-empirical mathematical model of Korsmeyer–Peppas was applied to study the kinetics of drug release from the yarns. The dosage form can be represented as follows:

(2) $$Q={\mathrm{kt}}^{n}$$

where Q is the amount of drug released at time t, K is the Korsmeyer–Peppas release rate constant, and n is the release exponent, which depends on the type of drug polydispersity, geometry, and transport. Depending on the release exponent, diffusional release mechanisms were classified as follows: n < 0.5, pseudo-Fickian diffusional behaviour; n = 0.5, Fickian diffusion; 0.5 < n < 1, non-Fickian diffusion; n = 1, case II transport (zero-order release); and n > 1, super case II transport [27].

#### 2.3.7. Antimicrobial Activity

*Staphylococcus aureus* (*S. aureus*) CECT240 (ATCC 6538p) and *Escherichia coli* (*E. coli*) CECT434 (ATCC 25922) strains were obtained from the Spanish Type Culture Collection (CECT, Valencia, Spain). The bacteria were thawed at 37 °C under agitation at 180 rpm for 24 h and were then diluted in a 1:1000 proportion in a Mueller–Hinton broth to obtain a concentration of 3–5 × 10^5^ colony-forming unit (CFU)/mL [28].

The antimicrobial activity of the fibrous yarns was studied using the disc diffusion method, following the protocols of the Clinical and Laboratory Standards Institute (CLSI) with some modifications [28,29]. Yarns of approximately 1 cm in length with and without CPX were deposited onto the Mueller–Hinton solid agar surface previously inoculated with 100 μL of *S. aureus* and *E. coli* (3–5 × 10^5^ CFU/mL). The samples were analysed in triplicate and incubated at 37 °C for 5 days. Inhibition diameters were measured after 1, 3, and 5 days, and the results are given as the mean ± S.D.

#### 2.3.8. Statistical Analysis

To detect differences between the fibre and yarn diameter, an unpaired *t* test was used using GraphPad Prism 8.0.1 software. For differences among halo sizes across the time course and polymer type, a Tukey multiple-comparisons 2 way ANOVA was carried out. Differences were considered statistically significant (*) when *p* ≤ 0.05.

## 3. Results

### 3.1. Morphological Characterization of Yarn Sutures

Upon yarn processing, different behaviours were observed for the different PHBVs. The PHBV with a higher valerate content (PHBV20) caused continuous breaking during the initial yarn formation, whereas PHBV with a lower 3HV content led to a more stable process, and little optimization was needed to collect meters of yarn, as was optimally the case for PHBV2. Under optimal processing conditions, the latter material could be processed at lower voltages and higher flow rates, suggesting better spinnability than their counterparts with higher 3HV content.

**Figure 2:**
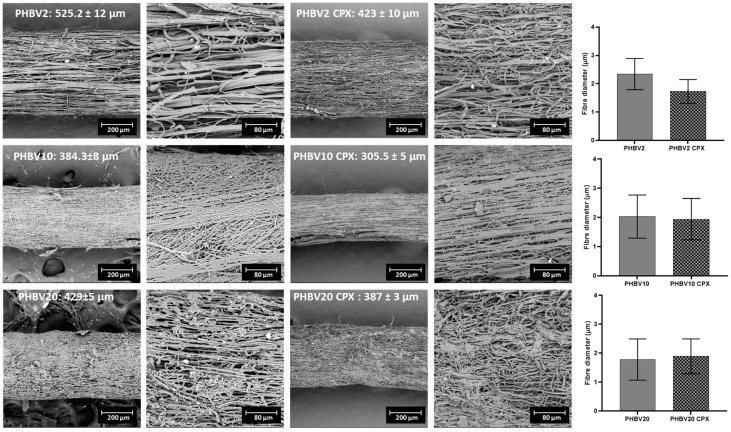
Scanning electron microscopy (SEM) micrographs (×300 and ×1000) of the PHBV yarns with 2% (PHBV2), 10% (PHBV10), and 20% HV content (PHBV20), and the CPX-PHBV-loaded yarns. Values in the images represent the average yarn diameter size ± S.D. On the right, the graphs illustrate the average fibre diameter ± standard deviation (S.D) of PHBV2, PHBV10, and PHBV20 with and without CPX. The scale bars are indicated in each micrographs.

The produced yarns were characterized by SEM to analyse the morphology of the fibres, as well as the overall diameter of the yarns. In terms of fibre diameter, the neat PHBV2, PHBV10, and PHBV20 and their counterparts containing CPX had a fibre diameter within the micrometer range (see Figure 2), with a preferential fibre orientation along the yarn collection. The presence of CPX in the yarns reduced the fibre diameter slightly compared to the neat PHBV yarns, although the results were not statistically significant for any of the samples (*p* > 0.05).

The overall diameter of the PHBV2, PHBV10, and PHBV20 yarns was ≈300–500 μm, as shown in Figure 2, with the PHBV2 yarn being thicker. In this case, the addition of CPX resulted in a reduction in the overall yarn diameter, which was statistically significant for all three PHBVs analysed. This phenomenon could be associated with a accumulative effect of the slight fibre size reduction discussed previously.

### 3.2. Thermal, Crystallinity and Molecular Characterization

**Figure 3:**
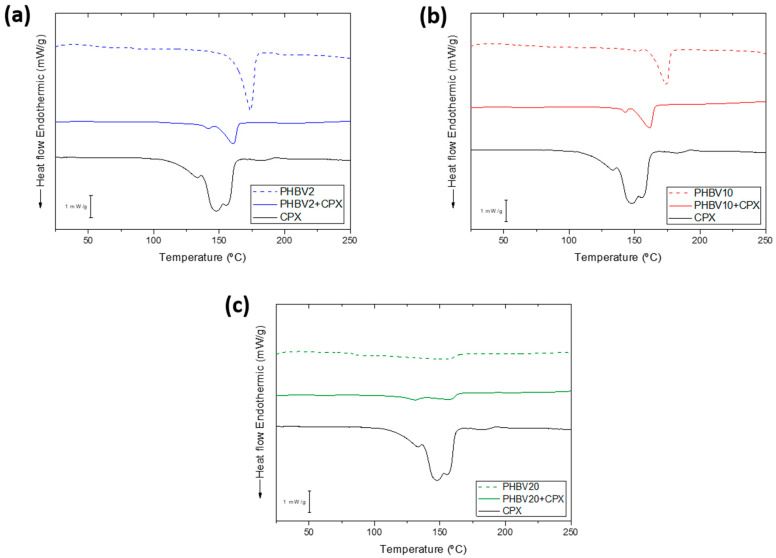
(**a**) DSC thermograms of neat PHBV2, PHBV2 + CPX, and pure CPX. (**b**) Neat PHBV10, PHBV10 + CPX, and pure CPX. (**c**) Neat PHBV20, PHBV20 + CPX, and pure CPX fibre yarn samples.

Yarns with and without CPX were analysed to obtain information on the crystallinity of the polymers in the presence of CPX. Figure 3 shows the DSC thermograms of the PHBV and PHBV-CPX yarns and pure CPX. The thermogram of the pure CPX (the black line present in Figure 3a–c) showed, in the range screened, a broad diffuse endothermic peak at around 150 °C, which was attributed to dehydration, in agreement with previous studies [30,31,32,33]. The melting point of the API occurs in the range of 255–257 °C. The thermograms of the neat PHBV2 and PHBV10 showed an endothermic peak associated with the melting point of the crystalline phase at 173 °C and 174 °C, respectively. The two polymers showed an enthalpy of fusion of 90.9 J/g and 70.5 J/g, respectively, suggesting the expected higher crystallinity of the PHBV2 [23]. As shown in Figure 3, the PHBV2-CPX and PHBV10-CPX yarns showed a decrease at both melting points, 161.1 °C and 161.8 °C, and enthalpy of fusion, 71.8 J/g and 61.8 J/g, compared to the neat biopolymer yarns, suggesting a decrease in polymer crystallinity and crystal size and/or density induced by the presence of the API. The thermograms of PHBV20 and PHBV20-CPX presented a weaker and broader melting behaviour, with maximum melting at 154.4 °C and 156.7 °C and enthalpies of fusion of 66.2 J/g and 50.21 J/g, respectively. The enthalpies of fusion of the drug-loaded samples included the small contribution of the shoulder at the lower temperature side of the melting peak, which might be associated with the CPX dehydration, hence even overestimating their crystallinity to some extent. As expected, the melting temperatures and enthalpies decreased with an increase in the 3HV content of the copolymers, which was ascribed to a decrease in crystallinity and crystal perfection [24,34,35]. Additionally, the PHBV-CPX peaks are usually broader and smaller than those of the neat PHBV yarns, which may also be attributed to the formation of crystals with lower perfection and broader size distribution, suggesting an overall deterioration of the crystalline morphology of the PHBV-CPX materials [35,36].

**Figure 4:**
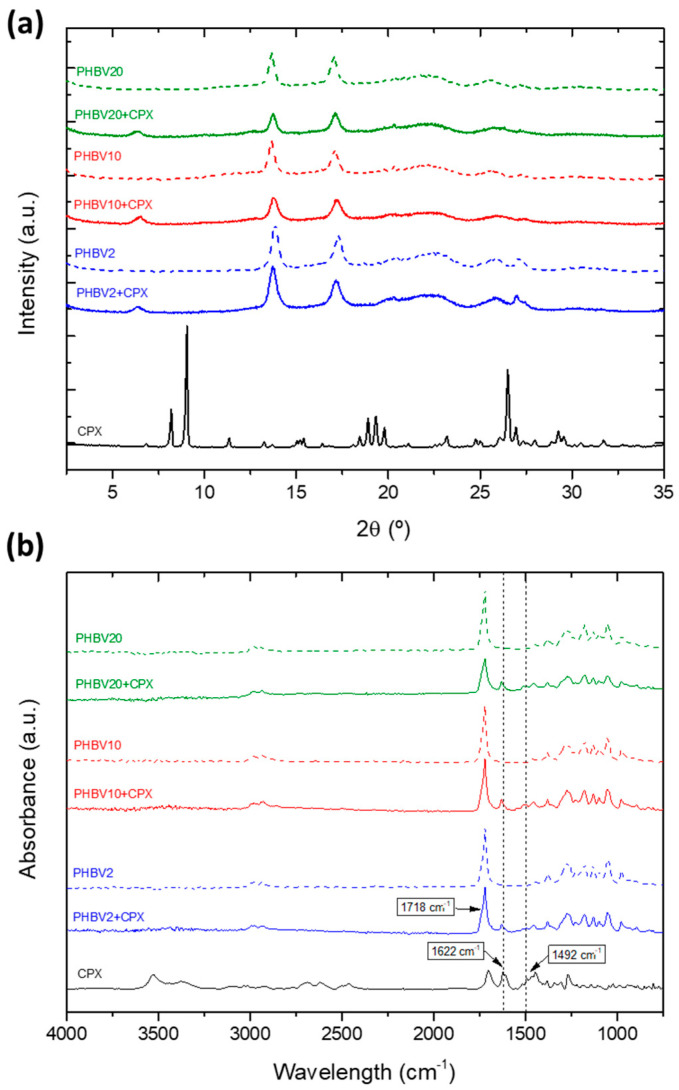
(**a**) WAXS spectra and (**b**) ATR-FTIR spectra of the pure CPX (black line), neat PHBV electrospun yarns (dashed lines), and CPX-loaded yarns. The dashed straight lines indicate the characteristic CPX peaks at 1622 and 1492 cm^−1^.

To further assess the crystalline morphology of the polymers and the drug in the yarns, the samples were analysed using WAXS (Figure 4a). The pure CPX exhibited the expected X-ray diffraction pattern of a crystalline material with three main sharp peaks at around 2θ at 8.2°, 9.0°, and 26.5°, in agreement with previous studies [32]. The X-ray patterns of PHBV with and without CPX showed the typical main peaks at 2θ of 13.7°, 17.0°, 25.0°, and 27.0° corresponding to the (020), (110), (121), and (040) lattice planes of the orthorhombic unit cells of PHBV, which are sharper for the lowest HV content sample, suggesting a more robust crystalline phase for the PHBV2, in agreement with previous studies [23,37,38]. The crystalline peaks seen in the CPX spectra were not identified in the PHBV yarns with CPX; however, a distinct new peak was observed at 6.4° in the spectra of all the CPX-loaded yarns, which was not present in the spectra of pure CPX. The appearance of this new peak might be related to the development of new ordered forms in the blend due to potentially strong interactions between the polymer and the drug [39].

Figure 4b shows a comparison of the ATR-FTIR spectra for pure CPX, neat PHBV, and CPX-loaded PHBV yarns. The yarns of the PHBVs showed their characteristic bands at 1718 cm^−1^, which were related to C=O stretching vibrations. The CH_3_ asymmetric and symmetric deformations were observed at 1452 cm^−1^ and 1379 cm^−1^, respectively, whereas C–O–C stretching was observed at 1278 cm^−1^, 1224 cm^−1^, and 1178 cm^−1^, in agreement with other studies [40]. For the CPX-loaded PHBV yarns, two non-overlapping characteristic peaks of the drug were found at 1622 and 1492 cm^−1^ (dashed straight lines in Figure 4a), associated with C=O and C-H stretching, respectively [41,42], thus confirming the presence of the drug in the yarns. As no spectroscopic alterations or new features ascribed to the degradation or reaction of the API with the polymer were unambiguously detected, the polymer-API blends were considered stable.

### 3.3. Process Loading Efficiency and In Vitro Release

Table 2 shows the results of the measured percentage of CPX loading found in the different electrospun yarns with regard to the drug placed in solution prior to electrospinning. The data suggest that the process efficiency is very high since more than 95% of the drug in the solution is encapsulated in the yarns.

**Figure 5:**
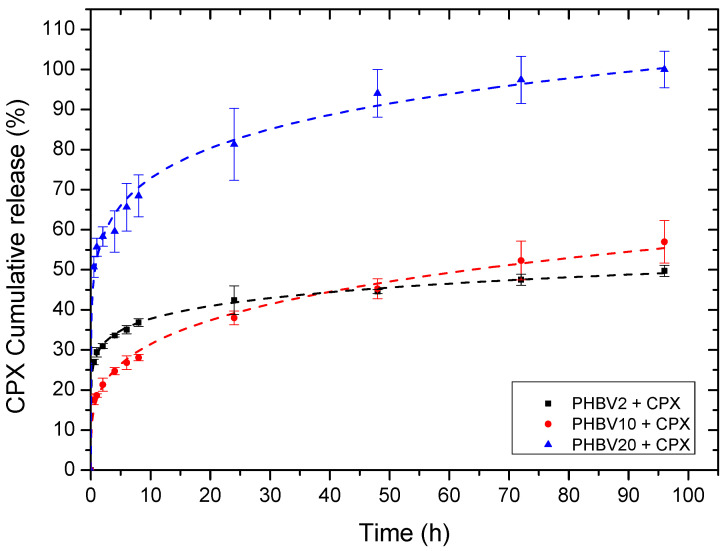
Drug release data comparing different yarns containing CPX. Dashed lines represent the Korsmeyer–Peppas fitting. Values are presented as mean ± SD (n = 3).

Figure 5 illustrates the cumulative drug elution from the yarns. The release curves were fitted to the Korsmeyer–Peppas model (Table 3). For all the samples, the values of the regression coefficient (*r*^2^) were high for the model, indicating a good fit and also suggesting that more than one type of drug release mechanism is taking place. In fact, the corresponding *n* values of the evaluated samples were all below 0.5, indicating that the drug release seemed to follow a pseudo-Fickian diffusional behaviour.

From a phenomenological viewpoint, the yarns made of PHBV2 exhibited a burst release followed by an arrested release after ca. 24 h, achieving a value of ca. 50% after 95 h. The initial burst release could be related to the presence of CPX on and near the fibre surface and can be quantitatively described by the *K* parameter (Korsmeyer–Peppas release rate) shown in Table 3. The arrested release after 24 h is likely attributed to the drug being more tightly bound inside the fibres and requiring a more tortuous path to diffuse out since the PHBV crystals are thought to arrest CPX elution.

For the PHBV yarn containing 10 mol% 3HV, a somewhat lower initial burst release was observed, followed by a somewhat faster sustained release reaching nearly 60% after 95 h. This initial burst behaviour is quantitatively characterized by the *K* parameter (Korsmeyer–Peppas release rate) since it presents the lowest value in comparison with the other two yarns. As the crystallinity of the PHBV2 sample was found to be higher than that of PHBV10, the initially higher burst release could be associated with the formation of crystals of the PHBV2 sample pushing out more of the drug towards the outside of the fibres.

The yarn with the highest number of 3HV units (20% mol) presented a burst release of approx. 70% after 10 h, followed by an arrested release of up to ca. 100% after 95 h. Such differing behaviour could be due to the more ill-defined crystalline phase suggested by the DSC and WAXS analysis, thereby facilitating faster drug elution.

### 3.4. Antimicrobial Activity

**Figure 6:**
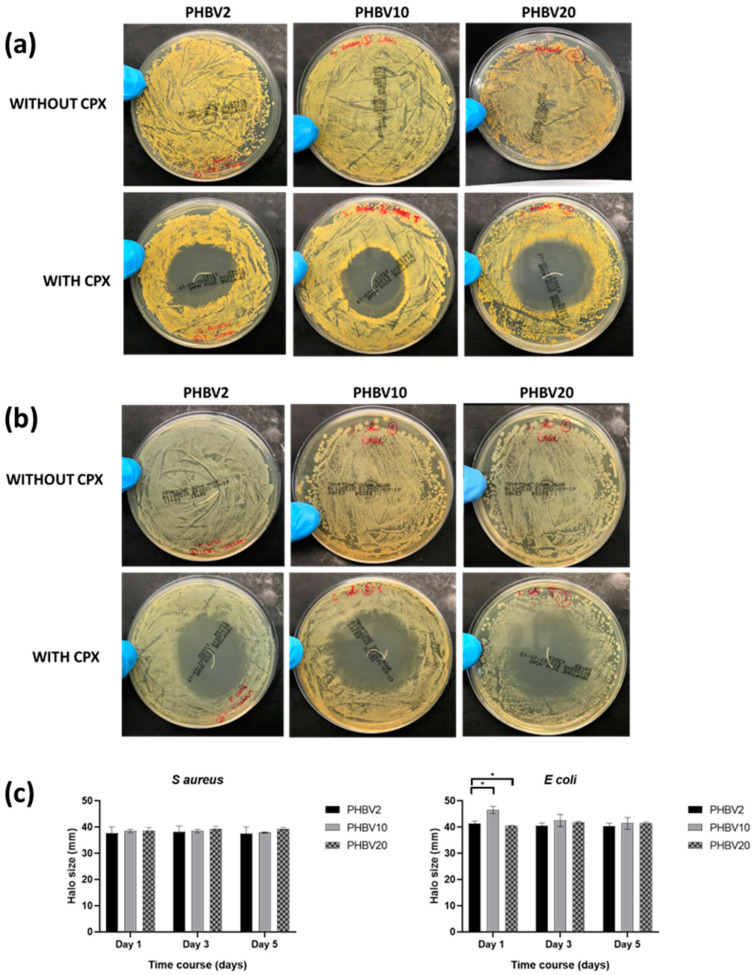
(**a**) Representative photographs of the growth inhibition of PHBV yarns with different HV contents (PHBV2, 10, and 20) with and without CPX against Staphylococcus aureus (*S. aureus*) after 5 days of culture. (**b**) Representative photographs of the growth inhibition of PHBV yarns with different HV contents (PHBV2, 10, and 20) with and without CPX against Escherichia coli (*E. coli*) after 5 days of culture. (**c**) Halo inhibition size of PHBV sutures with different HV contents (PHBV2, 10, and 20) with CPX against Escherichia coli (*E. coli*) and Staphylococcus aureus (*S. aureus*) after 1, 3, and 5 days of culture. Data are presented as the mean ± SD (n = 3). * indicates *p* value < 0.05.

Figure 6 shows the inhibition zones of the prepared yarns with and without CPX for both the Gram-positive (Figure 6a) and Gram-negative (Figure 6b) bacteria for up to 5 days. The yarns without CPX (PHBV2, PHBV10, and PHBV20) showed no inhibition zone for *S. aureus* and *E. coli*, indicating limited antibacterial effects (see Figure 6, micrographs without CPX). In the case of the CPX-loaded materials (PHBV2 + CPX, PHBV10 + CPX, and PHBV20 + CPX), very large inhibition halo zones were observed. An inhibition zone larger than 1 mm indicated a somewhat strong antibacterial effect at the site of action [6,43]. In the case of PHBV2 + CPX, the halo size was larger than 30 mm for both strains from day 1 to day 5, with a slight reduction towards the end of the experiment. PHBV10 + CPX and PHBV20 + CPX showed a similar trend, with halo sizes larger than 30 mm in all cases, especially for PHBV10+CPX in the *E. coli* strain, which showed significant differences during the first day of testing (Figure 6c, *p* < 0.05).

The effectiveness of loaded CPX materials as antimicrobial agents has been reported in the literature. For instance, in a study by our research group, patches made of blends of polyesters loaded with CPX reported an inhibition zone of approx. 5 cm after 24 h, proving that CPX-loaded fibres could efficiently act as antimicrobial multilayer systems [44]. Another study by Liu et al. reported the incorporation, characterization, and drug release of CPX in sutures by dip-padding, but no data were presented for in vitro antimicrobial testing [45]. Alternatively, sutures loaded with a fluoroquinolone family compound (levofloxacin) showed that 4% of loaded PEG/PLLA created a 2 cm inhibition zone after 24 h of drug release. In addition, 7 days after the drug-loaded sutures still provided bacterial inhibition, confirming that biologically active antibiotic was being released from the suture in an amount sufficient to eliminate surrounding bacteria [17], similar to what has been observed in this study.

### 3.5. Mechanical Properties

Tensile modulus (E), tensile strength (σ_b_), and elongation at break (ε_b_) were calculated from the stress–strain curves. Table 4 shows the mechanical properties of the yarns with and without CPX. For the neat yarns (without CPX), the tensile modulus tended to decrease with increasing 3HV content. Overall, the three materials exhibited brittle behaviour. The incorporation of CPX into the PHBV yarns tended to increase the tensile modulus and the tensile strength. This increase in yarn rigidity may indicate that the CPX acts as a reinforcing filler in the polymer matrix. Interestingly, other studies using electrospun drug-eluting sutures, more specifically anesthesia, reported that the suture’s strength decreased as the concentration of the drug in the suture increased. In this particular study, the loading of the drug in the suture varied from 5 to 22% [4]. In the present study, the use of ciprofloxacin at 20% loading seemed to stiffen the yarns. In another study conducted by Kamal et al. [46], planar electrospun PHBV containing cephalexin increased the elastic modulus and the tensile strength [46]. In another study, PLLA yarns showed a higher tensile stress compared to PLLA yarns loaded with curcumin [47]. The differences in mechanical properties among the studies can be influenced by many factors, such as the type of drug used, its dispersion throughout the polymeric matrix, crystallinity and hardness of the drug, interactions between the drug and the polymer, and the yarn morphology [1].

When comparing our materials to commercially available sutures, it is noteworthy to mention that these proof-of-concept yarns still need further development when it comes to their mechanical properties. Although the electrospun yarns presented herein led to optimum drug release, exhibited antimicrobial properties, and showed enhanced strength when the antibiotic was incorporated, they are still not as robust as the current commercially available sutures. For example, MonoMax^®^ sutures, which are commercial sutures made of poly-4-hydroxybutyrate, reported Young’s modulus of 485 MPa [48], which is lower than that of the PHBV-CPX yarns reported here. However, the elongation at break is generally higher in other reported suture materials than in the developed yarns herein [48,49,50]; thus, further optimization of the mechanical properties will have to be addressed in future studies.

On the other hand, when analyzing studies based on drug-eluting sutures made by electrospinning, the literature also reports mechanical properties below the cited comparable commercial standards [3,4,17]. Further studies will involve the use of polymer blends [51] and core-sheath strategies [52] to balance the mechanical properties.

In the current work, the influence of fibre yarn biodegradation under physiological conditions was not considered. However, other studies, such as the research conducted by E. I. Shishatskaya et al., indicate that the mechanical properties of filaments made from P(3HB) and P(3HB-co-3HV) experienced minimal alteration (approximately 90% of the initial value) after 30 days of degradation in the blood. These researchers concluded that PHAs could be considered safe and biodegradable materials suitable for long-term biomedical applications [53]. Moreover, the biodegradation process of the biodegradable polymers occurs via direct interaction between their hydrolyzable groups and the extracellular enzymes released by microorganisms. For this to happen, establishing a biofilm with enough free water in it is necessary so that the enzymes released by the microorganisms into the biofilm can reach and penetrate the polymer, thus reducing its hydrolysis and a subsequent reduction in the molecular mass [54]. Therefore, this process will not occur until the biofilm is well established, which requires an intimate and long interaction of the polymer with a substrate containing microorganisms capable of biodegrading this particular polymer. Thus, under usage and storage conditions, especially in the absence of free water, the shelf life of products made with biodegradable polymers is not limited. Additionally, the biodegradation process under natural conditions (i.e., not in conditions of organic waste treatment like industrial or domestic composting or anaerobic digestion) is a relatively slow process that can take years depending on the size of the samples and environmental conditions [55]. Therefore, the use of these materials in short-lived applications is not compromised by their biodegradability.

## 4. Conclusions

This study has shown, for the first time, the development and manufacture of electrospun drug-eluting yarns made of three PHBV polymers with varying 3HV contents loaded with ciprofloxacin hydrochloride. The materials were manufactured by needle-based electrospinning but adapted with a custom-designed funnel collector to synthesize in situ and continuously produced yarns based on ultrathin fibres. The morphology of the yarns for different PHBV grades showed cylindrical structures of approximately 300–500 µm made of ultrathin electrospun fibres with average diameters in the range of 1.95 ± 0.22 µm. The API appeared to be in an amorphous state within the yarns, and the polymer crystallinity was found to decrease with increasing HV content in the copolymers, which was correlated with the release profiles. Although the vast majority of the properties were similar across the different sutures tested, a faster burst release was found for PHBV CPX20, which could be more adequate to neutralize a potential post-surgery-related infection. The antibacterial activity of the different sutures was measured by halo size, suggesting a strong growth inhibition for two typical pathogenic strains after 5 days of culture, thus proving that they can be suitable for post-operation sutures in order to avoid surgical site infections. The yarns developed presented an increased stiffness when CPX was incorporated; however, when comparing the mechanical performance with other existing commercially available suture materials, it is considered that a somewhat higher elasticity can be more optimal for the application. Nevertheless, the materials developed present an interesting bio-based and anti-infective alternative to other conventional materials used today for wound healing and closure applications.

## Figures and Tables

**Table 1:** Optimal electrospinning parameters for the different tested solutions. It refers to the conditions used to generate electrospun yarns made of PHBV2, PHBV10, and PHBV20, with and without CPX.

Sample	Flow-Rate (mL/h)	Voltage V+/V− (kV)	Funnel to Yarn Collector Distance (cm)	Needle Distance to Funnel (cm)	Funnel Speed (rpm)	Yarn Collector (rpm)
PHBV2	12	10/−10	26.5	34	300	5
PHBV2 + CPX	10	13/−13	26.5	35.5	300	5
PHBV10	5	18/−18	25.5	34	300	5
PHBV10 + CPX	5	18/−18	28.5	34	300	5
PHBV20	3	15/−15	24.5	34	300	5
PHBV20 + CPX	5	15/−15	26.5	35.5	300	5

**Table 2:** The CPX loading efficiency (%) measured in different electrospun yarns.

Sample	CPX Loading in the Yarns (%)
PHBV2 + CPX	97.33 ± 2.42
PHBV10 + CPX	95.50 ± 7.50
PHBV20 + CPX	96.54 ± 4.43

**Table 3:** Model parameters of CPX release profiles obtained from the Korsmeyer–Peppas model.

Korsmeyer-Peppas			
Sample	K	n	r^2^
PHBV2 + CPX	28.88	0.12	0.99
PHBV10 + CPX	17.68	0.25	0.99
PHBV20 + CPX	52.56	0.15	0.99

**Table 4:** Mechanical properties of ciprofloxacin-loaded PHBV sutures. Data represent the mean ± S.D. (n = 5).

Sample	E (MPa)	σ_b_ (MPa)	ε_b_ (%)
PHBV2	660 ± 131	8.4 ± 1.8	14.3 ± 1.6
PHBV2 + CPX	996 ± 224	14.5 ± 3.2	11.0 ±0.5
PHBV10	575± 281	10.1± 2.6	14.8 ± 5.1
PHBV10 + CPX	1099 ± 204	13.6 ± 2.0	14.7 ± 9.1
PHBV20	438 ± 126	8.5 ± 2.4	10.6 ± 3.2
PHBV20 + CPX	731 ± 334	11.1 ± 1.8	16.5 ± 6.3

## Data Availability

Data are contained within the article and supplementary materials.

## Supplementary Materials

The following supporting information can be downloaded at: https://www.mdpi.com/article/10.3390/pharmaceutics16020220/s1, Video S1: Electrospun yarn-producing device operating.
